# Targeted action to increase inclusion at the Wellcome Sanger Institute

**DOI:** 10.7554/eLife.94732

**Published:** 2024-02-21

**Authors:** Saher Ahmed, David J Adams, Muzlifah Haniffa, Aidan Maartens, Maria Augusta Arruda

**Affiliations:** 1 Wellcome Sanger Institute Hinxton United Kingdom; 2 Genome Research Limited Hinxton United Kingdom; 3 Newcastle University Newcastle upon Tyne United Kingdom; 4 Newcastle Hospitals NHS Foundation Trust Newcastle upon Tyne United Kingdom; 5 Brazilian Biosciences National Laboratory, National Center for Research in Energy and Materials (LNBIO-CNPEM) São Paulo Brazil

**Keywords:** research culture, equity, diversity, inclusion, careers in science, None

## Abstract

The Sanger Excellence Fellowship has been established to increase the representation of researchers with Black-heritage backgrounds at a leading research centre in the UK.

## Introduction

The racial inequalities observed in societies across the globe are mirrored in the scientific community in the UK, especially at more senior levels, where there continues to be a lack of Black, Asian and Ethnic Minority scientists in leadership positions (Arday, 2018). In the UK, for example, although Black students are overrepresented in the undergraduate population, they are underrepresented among postgraduates and junior research staff (Gibney, 2022; RSC, 2023). Across academia, of the 22,855 professors in the UK, just 160 are Black, and the representation of other minority groups also declines with seniority. This lack of role models in senior positions discourages younger Black scientists from pursuing careers in research.

As well as being inherently unjust, underrepresentation also hinders scientific progress because it means that the scientific enterprise is not taking full advantage of the diversity of skills and lived-experiences available to it (Urbina-Blanco et al., 2020). Furthermore, there is evidence for a “diversity-innovation” paradox whereby historically marginalised groups have less successful careers despite displaying higher rates of innovation (Hofstra et al., 2020). Despite these negative effects, there have been few targeted interventions to drive meaningful change in academia; the resulting frustration and disenfranchisement of marginalised communities is illustrated by clinical psychologist Sanah Ahsan’s redefinition of EDI as ‘Endless Distraction and Inaction’ (Ahsan, 2022).

**Figure 1.**
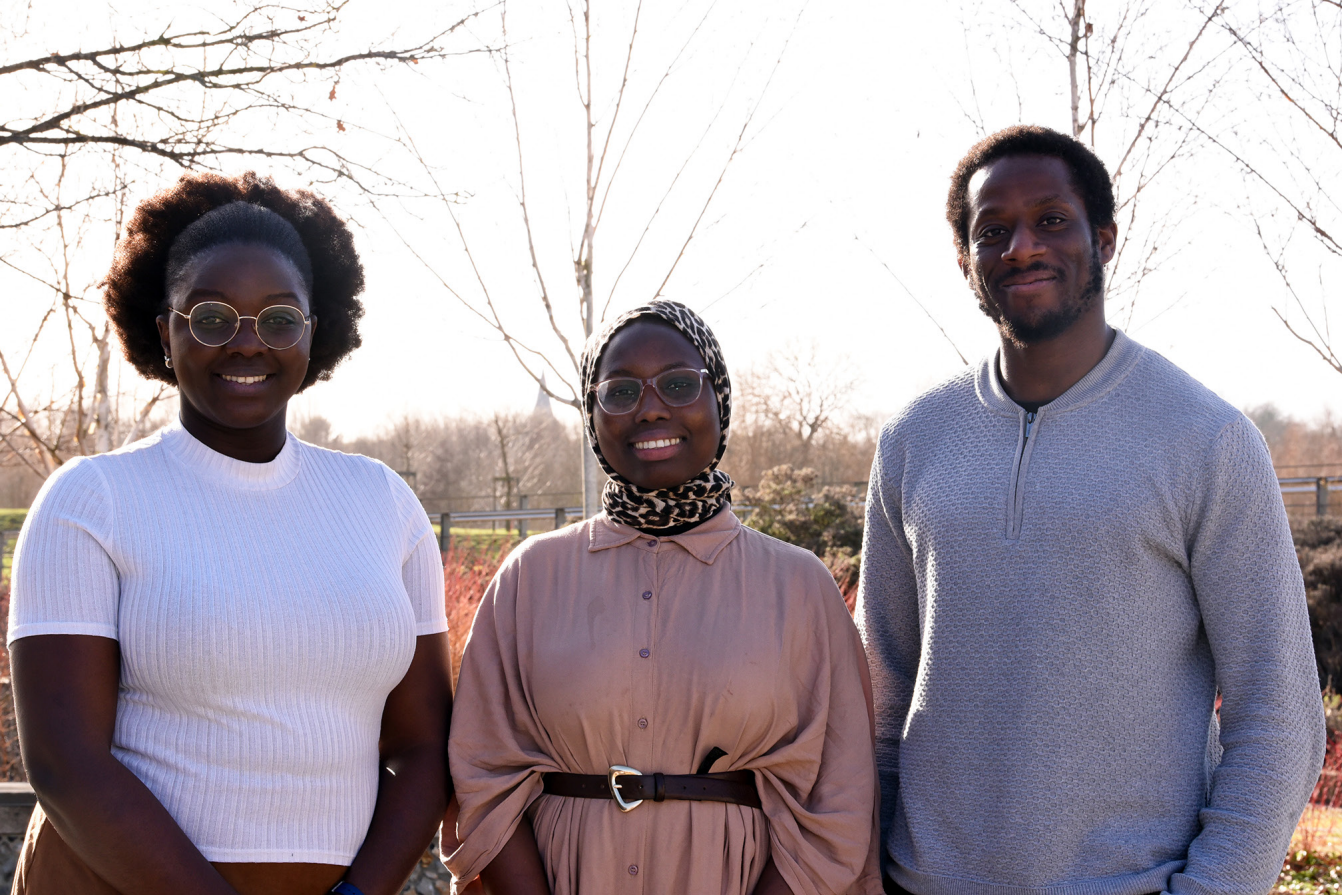
Recipients of the first three Sanger Excellence Fellowships: Kudzai Nyamondo (left), Oumie Kuyateh (middle) and Ore Francis.

Here, we describe a scheme to address Black underrepresentation among early-career researchers at the Wellcome Sanger Institute in the UK. The recipients of the first three Sanger Excellence Fellowships were announced in October 2022 (Figure 1), three were awarded in 2023, and the third round of applications opened in February 2024 (see Box 1). Our scheme is one of a small number of schemes aiming to tackle underrepresentation in science in the UK (see Table 1 for other examples). Of note, the Royal Society is also running a pilot scheme that offers four-year Career Development Fellowships to Black postdoctoral researchers (applications now closed), while Wellcome has just announced a scheme to fund researchers of Black, Bangladeshi and Pakistani heritage in the UK.

Credit: Wellcome Sanger Institute.

Box 1. **The Sanger Excellence Fellowship**

The Sanger Excellence Fellowship scheme is open to applicants who identify as being from a Black-heritage background, and have an undergraduate degree and PhD or equivalent from institutions in the UK and Ireland. The scheme was established in 2021, and the first pre-call for applications was announced in January 2022. Initially, we planned to fund two fellowships, but support from Cancer Research UK (CRUK) enabled us to fund a third fellowship with a focus on cancer. Fellows can work in any of the six research programmes at the Sanger (Cancer, Ageing and Somatic Mutation; Cellular Genetics; Generative and Synthetic Genomics; Human Genetics; Parasites and Microbes; Tree of Life). Our website (https://www.sanger.ac.uk/about/equality-in-science/sanger-excellence-fellowship/) contains more information on the fellowship (including a list of the members of the scheme’s Advisory Group).

The first three fellows were announced in October 2022 (Figure 1), another four fellows were selected last year, and the call for applications for the third round of fellowships is being launched this month (February 2024). More information on the first seven fellows is below, and support from the Medical Research Council means that we should be able to once again fund four fellowships in this round.

**Kudzai Nyamondo** holds a PhD in cancer sciences from the University of Glasgow, and works in the Cancer, Ageing and Somatic Mutation programme. She aims to understand the timelines and trajectories of blood cancers, both to advance our understanding of cancer evolution and to accelerate early detection and intervention strategies. Dr Nyamondo’s fellowship is jointly funded by Cancer Research UK and the Wellcome Sanger Institute.

**Oumie Kuyateh** has a PhD in evolutionary biology from the University of Edinburgh, and works in the Parasites and Microbes programme. She is interested in the development of the respiratory microbiome in early life and its regulation by environmental factors, and hopes her approach will aid in the diagnosis and treatment of respiratory infections.

**Ore Francis** completed his PhD in biochemistry in the University of Bristol, and works in the Tree of Life programme, which aims to genetically map all known species in Britain and Ireland. Dr Francis aims to use sequence predictions of proteins in eukaryotes to understand how they cooperate to make life possible.

**Michael Ansah** is investigating genetic diversity, pathogenicity and therapeutic targets in *Acanthamoeba* (Tree of Life programme).

**Samantha Jumbe** is working on somatic mutations in the aetiology of autoimmune disease (Cancer, Ageing and Somatic Mutations programme).

**Onalenna Neo** is using AMR capture probes to unveil the dynamics and mechanisms of resistance transmission in diverse environments (Parasites and Microbes programme).

**Mona Suleiman** is working experimental evolution in the parasitic worm *Strongyloides* to map ivermectin drug resistance in the lab and field (Parasites and Microbes programme).

**Table 1.** Examples of current schemes tackling underrepresentation in science in the UK.

General schemes
Cowrie Scholarship Foundation Undergraduate: Supports disadvantaged Black British students at UK universities.
Royal Society Career Development Fellowship Postdoctoral: Four-year fellowships to retain researchers from underrepresented backgrounds in science, technology, engineering and mathematics.
Health Data Science Black Internship Programme Workforce: Eight-week paid internships to tackle the underrepresentation of Black people within the health data science sector.
Wellcome: Targeted funding for researchers of Black, Bangladeshi and Pakistani heritage Postdoctoral and higher: Awards of up to £200,000 to support researchers of Black, Bangladeshi and Heritage in the UK to stay in research and advance their careers. Total funding is £20 million over four years.
Schemes at specific institutions
Stormzy Scholarship for Black UK Students (University of Cambridge) Undergraduate: Scholarships to provide financial support for Black UK students at Cambridge.
Excel in Science Internship Programme (University of Nottingham) Undergraduate: Paid internships to give budding researchers the opportunity to gain hands-on research experience at Nottingham.
Surrey Black Scholars Programme (University of Surrey). Undergraduate and postgraduate: Programme to provide Black British students at Surrey with the resources, support and environment necessary to achieve excellence and pursue rich and rewarding careers after graduation.
James McCune Smith PhD Scholarships (University of Glasgow) Postgraduate: Scholarships to fund Black UK students to undertake PhD research at Glasgow.
Academic Futures Scholarship (University of Oxford) Postgraduate: A series of scholarship programmes to address underrepresentation and help improve equality, diversity and inclusion among graduate students at Oxford.
Cancer Research UK: Black Leaders in Cancer PhD Scholarship Programme (CRUK City of London and Cambridge Centres) Postgraduate: Programme aimed at students from Black heritage backgrounds pursuing a PhD in cancer-related fields.

We recognize that our programme is a work in progress, and there is still a great deal to do to improve representation at the Sanger, and in the scientific community in the UK more widely. But by developing a programme that centres the voices of Black academics, and is continually guided by feedback, we hope we can enthuse and inspire other organisations to consider how they can take practical steps to reduce inequalities in scientific research and wider academia.

## Developing a strategy to increase inclusion

**Figure 2.**
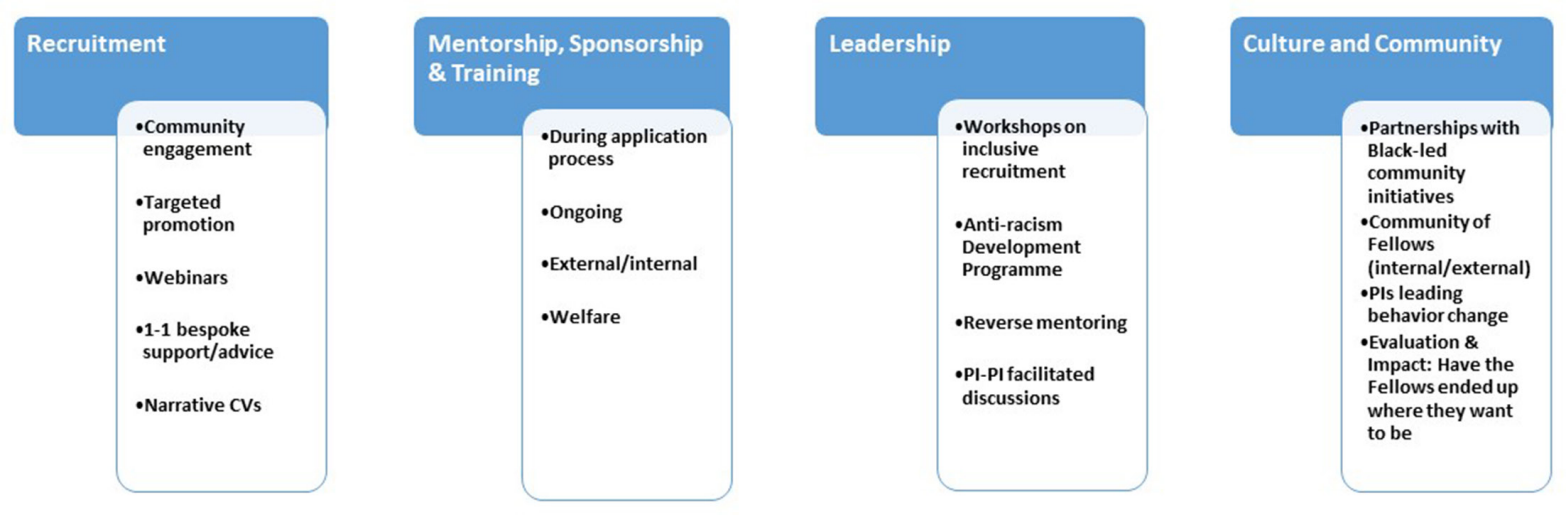
Introducing the Sanger Excellence Fellowships involved various changes at the Wellcome Sanger Institute. The changes to our recruitment process, and the additional mentorship, sponsorship and training available to the new fellows, are described in the main text. The leadership of the institute were involved in various workshops and related activities. There was also a sustained effort to change the culture at the institute by working in partnership with grassroots community organisations to run workshops and events, and provide mentoring and sponsorship opportunities.

The Wellcome Sanger Institute is a world-leading research centre that conducts research in many areas of genetics and genomics. It is based at the Wellcome Genome Campus just south of Cambridge in the UK, and employs over 1,200 staff. The institute aims to foster an inclusive culture where everyone can thrive and diversity is celebrated, and as part of our overall strategy for equity, diversity and inclusion, we have been developing a race equity strategy in collaboration with our community (Figure 2). This strategy relies on quantitative and qualitative insights, bolstered by self-assessment processes. For instance, we are committed to the Advance HE Race Equality Charter which aims to help higher education institutions work towards improving the representation, progression and success of Black, Asian and Minority Ethnic staff and students.

Our race equity strategy aims to support underrepresented groups entering the Sanger at any level and in any capacity, in a number of ways: by building competency and literacy around discussing race equity (for instance by hosting campus-wide events and workshops); by using data to inform strategic planning; and by embedding anti-racist approaches in the way we work.

As part of this strategy, we initiated a series of discussions about how we could support underrepresented researchers as they progressed from undergraduate to postgraduate to postdoc to established researcher. Deciding which stage of this academic career ‘pipeline’ to target would be a very important decision. We also wanted to disrupt the traditional academic notion of ‘research excellence’ and its associated measures of success, conscious that the odds of succeeding in science are influenced by a number of factors other than a talent for research, such as biases related to gender, race, socioeconomic status and other characteristics (Liu, 2020). Our efforts were greatly helped by our Advisory Group, which included Principal Investigators (PIs) from the Sanger, Black leaders from various UK institutions, representatives from charities and funders, and specialists with experience in promoting inclusivity in science. This group also included three of the authors (SA, DJA and MAA). Following extensive discussions, we eventually coalesced around three specific aims.

First, we decided to focus on Black underrepresentation, rather than underrepresentation more broadly. We made this decision by following the data – people from Black-heritage backgrounds are the most underrepresented group at the Sanger among both staff and students, and the same is true for the wider scientific community in the UK (Gibney, 2022; Arday et al., 2022).

Second, we decided to focus our efforts on postdoctoral researchers. The academic career pipeline in science begins in schools (where diversity is highest) and ends with positions of leadership (where diversity is lowest). Postdoctoral research sits halfway along the pipeline, and is a crucial career stage that precedes a researcher becoming independent and running their own research group (or progressing to other careers in or outside universities). By focussing on this career stage, we aimed to ensure Black scientists who have completed PhDs have an opportunity to remain in scientific research and fully realise their potential.

Third, while the Sanger Institute already offered three-year fellowships for postdoctoral researchers that included financial support for research and travel, we decided that the new scheme should also include non-financial support. Therefore, in addition to receiving all the benefits of a regular fellowship at the Sanger, holders of the new fellowships receive additional mentorship, sponsorship and targeted training.

By linking with external organisations, we aim to foster connections between our fellows and funders. For instance, we arranged for the fellows to present their work at the Wellcome headquarters in London, and to meet with grant managers there in order to learn more about funding opportunities, and our Sanger–Cancer Research UK fellow has access to a bespoke mentorship programme. We facilitate links to grass-roots communities such as Black in Cancer and the Black Women in Science Network to promote networking. We provide sponsorship, in terms of putting our fellows forward for career development opportunities, and arranging additional training opportunities. We have also formed connections with more formal groups such as the MRC’s Black in Biomedical Research Advisory Group that the fellows can utilize for mentorship and support, and we are currently in discussions with other organisations to promote networking opportunities.

Throughout the development process, we kept in mind that the scheme was not just about the new fellowships. What we really wanted to do was break apart the usual ways of working and thinking, to challenge ourselves in terms of how science should be run. For example, while we were developing the programme, we ran a workshop of facilitated discussions with Leading Routes, an organisation aiming to prepare the next generation of Black academics, to help PIs at the Sanger explore topics of racial equity. We wanted to deconstruct the notion of research excellence and to really think about potential versus access to opportunity. Central to our approach was moving out of our comfort zone and embracing that discomfort through safe and supported dialogue and conversation.

## The recruitment process

Once we had decided what shape the fellowship should take, a key challenge was to attract applications. To do this, we created a bespoke application process that was centred on outreach and connecting with communities.

We built partnerships with grassroots communities such as Black in Cancer and the Black Women in Science Network, who helped us to promote the fellowships to their members. We also worked with recruiters who specialise in reaching to diverse candidate pools (for example, https://wearediverse.io/). An extended social media campaign helped raise awareness of the scheme, and we also ran a series of live webinars and live Q&As for potential applicants to demystify academic pathways – for instance, exploring what it means to do a postdoctoral fellowship at Sanger – and all steps of the application process.

We also had to completely rethink the rest of recruitment process. At the core of all applications was the project, designed by the applicants with guidance and mentorship from potential supervisors. This process itself was part of the “sponsorship” of the candidates, as the supervisors connected them into their research networks.

This process involved a series of one-to-one discussions between supervisors and candidates to develop the research proposal, allowing candidates to learn about the design of successful research plans from world-leading scientists. Many candidates will not have been offered this kind of bespoke support before, and other postdocs do not typically develop their own research proposals in a similar way.

A novel aspect of the application process was a move to narrative CVs, which are based on the Resume for Researchers developed by the Royal Society (see Fritch et al., 2021 for a report on how narrative CVs can reduce biases and advice on how to design such CVs). In their narrative CVs, we ask candidates to highlight their contributions to various aspects of research: the generation of knowledge, including all the outputs of their work, not just research papers; the development of individuals, including mentorship, collaboration and education; contributions to the wider research community (such as reviewing, organising events or committee work); and contributions to broader society (such as engaging with the public, public bodies or the private sector). We also provide candidates with space to detail any personal challenges they had to overcome to get to where they are today. At the heart of the process is trying to unpick potential versus access to opportunity.

Together, we strongly believe this approach provides a broader view of research achievements and potential than standard CVs. We hoped to use it mitigate our own biases in terms of what constitutes research excellence, and thus identify those with the highest potential to succeed in the research environment.

## Lessons for the future

Looking forward, we are keen to expand the scheme, and to explore new ways to make the Sanger Institute more inclusive, so that all talent can genuinely thrive. One idea we are considering is reverse mentorship with the Excellence Fellows, ensuring that the new fellows can provide feedback directly to the institute leadership.

While it is still too early to assess the overall success of the scheme with our first fellows still in the early stages of their time at Sanger, we will track their careers going forward and seek direct feedback on how the scheme could be improved. Additionally, we commissioned an external evaluation of the scheme that engaged both successful and unsuccessful applicants (Nexus Evaluation, 2023). We were heartened that the fellowship was seen as uniquely designed positive action, and have taken in the comments for how it could be improved as we go forward.

We also want to be looking further along the academic career pipeline – how can we innovate to ensure that talented minoritized postdoctoral researchers who wish to run their own labs are retained to independence? This is a vital challenge.

Our blueprint for change is just one scheme within a complex landscape of UK academia, and poses a question for funders: how can we work together to guarantee the sustainability of current and future initiatives in a way that supports historically marginalized researchers at all stages of their careers? The ultimate aim is to ensure UK science flourishes, and that cannot happen unless all voices are empowered to participate in driving our science.
